# One case report of epilepsy and rapidly progressive cognitive impairment after levofloxacin treatment

**DOI:** 10.1186/s12888-023-05425-0

**Published:** 2023-12-07

**Authors:** Zhan Su, Guimei Zhang, Yanxin Shen, Xiangting Li, Zixun Wang, Haining Zhang

**Affiliations:** Department of Neurology and Neuroscience Centre, the First Hospital of Jilin University, Changchun, China

**Keywords:** Levofloxacin, Seizures, Rapid Progressive cognitive impairment

## Abstract

**Objective:**

To report a case of seizure and rapidly progressive cognitive impairment 20 min after intravenous administration of levofloxacin.

**Case summary:**

A 56-year-old woman was admitted to hospital with episodic unconsciousness and unresponsiveness. About 4 days ago, she experienced a loss of consciousness, fell to the floor, and yelled for 2 min, 20 min before the first intravenous dose of levofloxacin. The patient developed symptoms of cognitive impairment after the seizure. Levofloxacin is a synthetic third generation fluoroquinolone used to treat various infectious diseases. Upon admission, the patient was conscious and unresponsive. After 11 days of symptomatic and supportive treatment, the patient was discharged from the hospital with cognition restored to baseline level and no recurrence of seizures 10 months after discharge.

**Discussion:**

Epilepsy is a rare adverse reaction to levofloxacin treatment. The patient in this case had infection-related signs before the onset of the disease, and the disease progressed rapidly with fluctuating changes. After ruling out degenerative, infectious, toxic, and autoimmune causes, the patient’s symptoms may be attributed to levofloxacin, and this is the first case of seizure and rapidly progressive cognitive impairment after levofloxacin injection reported in the literature. Clinicians should be aware that unexplained, rapidly progressing cognitive impairment with infection-related signs before onset may be a rare side effect of antibiotics.

**Supplementary Information:**

The online version contains supplementary material available at 10.1186/s12888-023-05425-0.

## Introduction

Levofloxacin is a synthetic third-generation fluoroquinolone with common indications including community-acquired pneumonia, skin and soft tissue infections, urinary tract infections, and sinusitis. With the popularity of its use, reports of adverse drug reactions have increased. Most of the adverse events associated with levofloxacin were mild, with the most common adverse events being nausea, headache, diarrhea, insomnia, constipation, and dizziness. The overall incidence of fluoroquinolone-related central nervous system disease is 1–3% [1], and epilepsy, a rare adverse drug reaction, has a seizure rate of 0.1-1% [2, 3].However, cases of rapidly progressing cognitive impairment as an adverse reaction to fluoroquinolones have not been reported. Therefore, the purpose of this report is to present a case of a person who developed seizures after levofloxacin injection and rapidly progressive cognitive impairment after regaining consciousness. After excluding degenerative, infectious, toxic, and autoimmune causes, the chronology of the patient’s onset and recovery suggests that levofloxacin was responsible for the manifestations of cognitive impairment in this patient.

## Case description

We describe a 56-year-old female patient who was admitted to the hospital with episodic unconsciousness and unresponsiveness for 4 days. The patient presented with low fever, sore throat, cough, and white sputum in the week prior to admission. She received her first intravenous infusion of levofloxacin 4 days ago, after which the patient quickly developed symptoms of discomfort and immediately stopped using levofloxacin. After 20 min, the patient briefly lost consciousness and continued to shout for about 2 min, while her eyes rolled up on both sides and she was unable to stand or respond. The patient was then taken to the emergency room. After regaining consciousness, the patient developed severe cognitive impairment. Her reflexes are slower than before, her memory is declining, her reactions are slow. She has difficulty performing activities of daily living, such as buttoning shirts or putting on pants. She began to rely on her husband for all activities of daily living, including bathing, dressing, and eating. In addition to the above symptoms, she had mild drowsiness, weakness of limbs, cyanosis of the lips, mild dysarthria, tremors, fever and abdominal pain. She was then transferred to a cognitive impairment center.

Her medical history includes hypertension, lacunar infarction, lumbar spondylolisthesis, giant cell tumor of tendon sheath of dorsalis pedis, and pulmonary tuberculosis. The patient had no history of epilepsy and denied drinking alcohol. Her family confirmed that she had no recent history of falls, food poisoning or other exposures that might have caused her symptoms.

In the emergency department, the patient was confused and depressed, and neurological tests showed her reflexes were weakened. After entering the neurology department, he underwent a thorough neurological examination. Her initial vital signs were heart rate 73 beats per minute, blood pressure 144/89 mm Hg, body temperature 36.4 °C, and respiratory rate 18 beats per minute. Peripheral blood oxygen is 94%. Her summary mental state test score was 11/30, and she was unable to perform computational tests and reproduce graphics(Figure S1). On physical examination, the main findings were dysfunction of higher cortex, mainly manifested as decreased orientation, memory, and calculation. In addition, the patient also had blurred speech, short step length, wide-base gait, drunken gait, horizontal and vertical nystagmus.

Her blood work came back normal. It includes blood counts, biochemistry, vitamin B1 and B12 levels, homocysteine levels, and normal liver and kidney function. Thyroid function tests were normal. CSF cells, sugars and lactic acid were in the normal range, and CSF protein was 0.81 g/L. MRI scans of the brain did not indicate acute changes. A chest CT scan suggests mild pneumonia. Ekg is normal. She completed lumbar puncture, cerebrospinal fluid autoimmune encephalitis antibodies, paraneoplastic neuronal antibodies, and normal or negative TBA screening on day 1 of admission and day 4 of onset. On the second day of admission and the fifth day of onset, EEG showed that there were slightly more sharp waves in bilateral frontal area and bilateral temporal area during the interattack period, and the full and slow waves were not distributed synchronously. Over the next few days, the patient’s family reported that his symptoms went on and off, with dizziness and blackness two or three times during the night. Her treatment included levetiracetam antiepileptic therapy, ginkgo diterpenoids for cognitive therapy,oxygen inhalation, nutrient and fluid input management, and supportive therapy. On the 6th day after admission, the patient’s cognitive function and strength were significantly improved, and his gait, memory, orientation and calculation were significantly improved compared with those before physical examination. The patient was discharged on day 11 with cognitive function restored to baseline. The patient was still treated with levetiracetam after discharge.

Patients still come to our center regularly for follow-up consultations. We reviewed the patient’s complete cognitive scale at 1 and 12 months, and her MMSE recovered to 17 (1 month) and 22 (12 months) (Figure S2, S3). Until her last consultation 12 months after admission, her seizures did not recur and levetiracetam was discontinued.

## Discussion

Fluoroquinolones are broad-spectrum antibiotics, and the most serious reactions caused by this class of drugs include toxicity of the central nervous system, cardiovascular system, and musculoskeletal system [4]. Levofloxacin-induced neurotoxicity is A known but rare side effect, and current reports include dizziness, headache, epilepsy, sensory disturbances, peripheral neuropathy, etc [5–7]. Possible mechanisms include inhibition of gamma-aminobutyric acid A (GABA-A) receptors and activation of N-methyl-D-aspartate (NMDA) receptors [8]. In this case, the transient loss of consciousness and cognitive impairment experienced by the patient are highly likely to be a serious adverse drug reaction to levofloxacin. In addition, the patient’s cognitive function and strength improved significantly on the sixth day after admission, as there was a clear chronological sequence between the onset of her symptoms and the application of levofloxacin. Moreover, the patient only received conventional and antiepileptic therapy during admission, which was insufficient to explain her cognitive improvement. The neurologist believed that the patient’s cognitive improvement was the result of levofloxacin metabolism. To our knowledge, this is the first case report of seizures and reversible fast-progressive cognitive impairment occurring 20 min after levofloxacin administration. We conclude that the hypothesis of adverse drug reactions in rapidly-progressive cognitive impairment of unknown cause cannot be ignored.

### Electronic supplementary material

Below is the link to the electronic supplementary material.

Supplementary Material 1

## Data Availability

The data that support the findings of this study are available from the First Hospital of Jilin University but restrictions apply to the availability of these data, which were used under license for the current study, and so are not publicly available.However, with the permission of the First Hospital of Jilin University, information can be obtained from the corresponding author upon reasonable request.
