# Supplementary material for: One case report of epilepsy and rapidly progressive cognitive impairment after levofloxacin treatment
Source: BMC Psychiatry. 2023 Dec 7;23:918. doi: 10.1186/s12888-023-05425-0 (PMC10704816; doi:10.1186/s12888-023-05425-0)
Supplement: Supplementary file 1 — Supplementary Material 1 [file 12888_2023_5425_MOESM1_ESM.docx]

Supplementary Materials

### One case report of epilepsy and rapidly progressive dementia after levofloxacin treatment

Zhan Su^1^, Guimei Zhang^1^, Yanxin Shen^1^, Xiangting Li^1^, Zixun Wang^1^and Haining Zhang^1^*

1 Department of Neurology and Neuroscience Centre, the First Hospital of Jilin University, Changchun, China.; suzhanzi@163.com

*Correspondence: zhanghaining@jlu.edu.cn; Tel.: +86 431 88782378

**This PDF file includes:**

Figs. S1 to S3


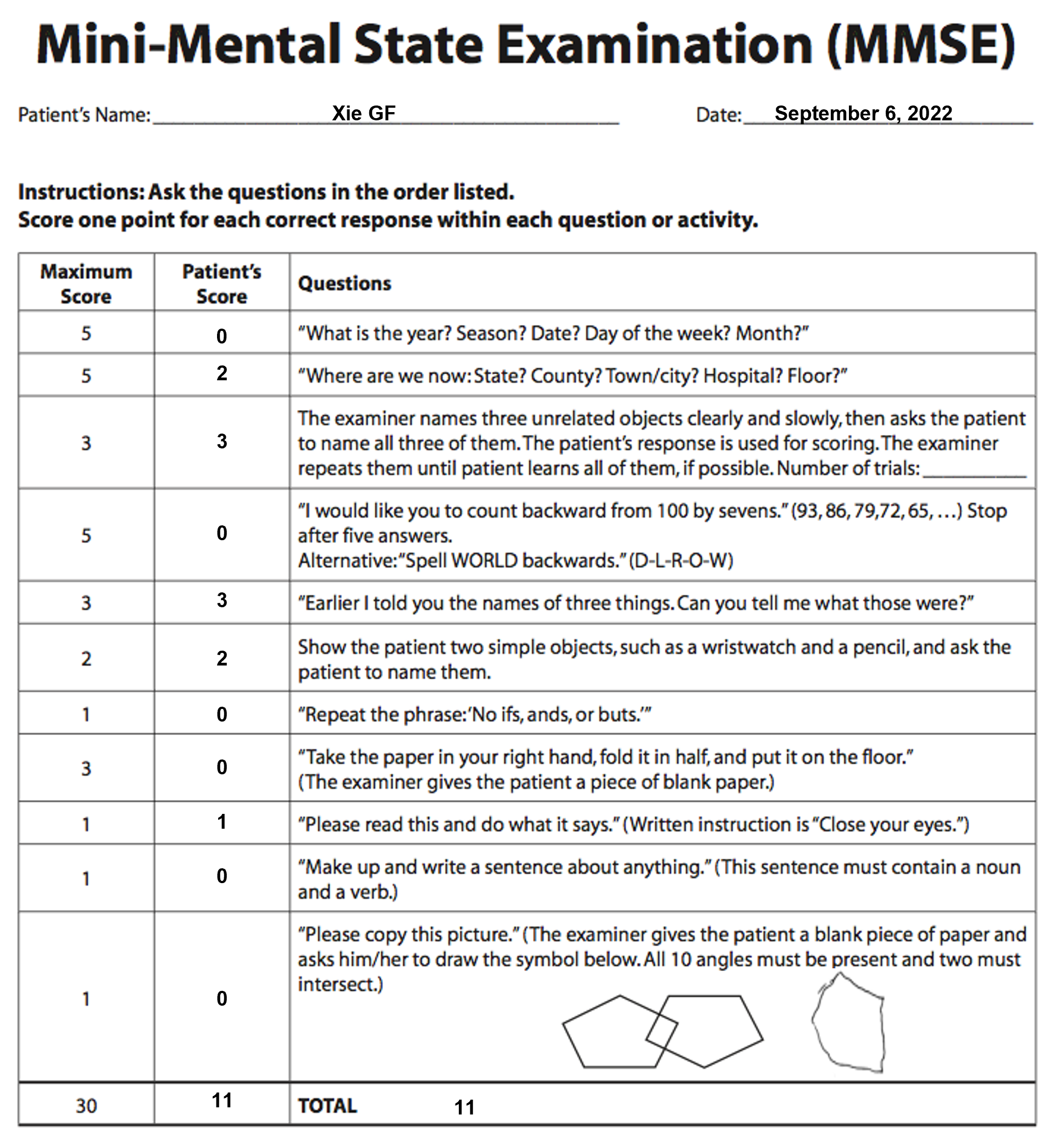


**Figure S1.** On the fourth day of onset (the first day of admission), the patient's MMSE score was 11( 2 points for orientation; 3 points for memory; 0 points for attention and calculation ability; 3 points for recall;3 points for language ability; and 0 points for copying graphs).


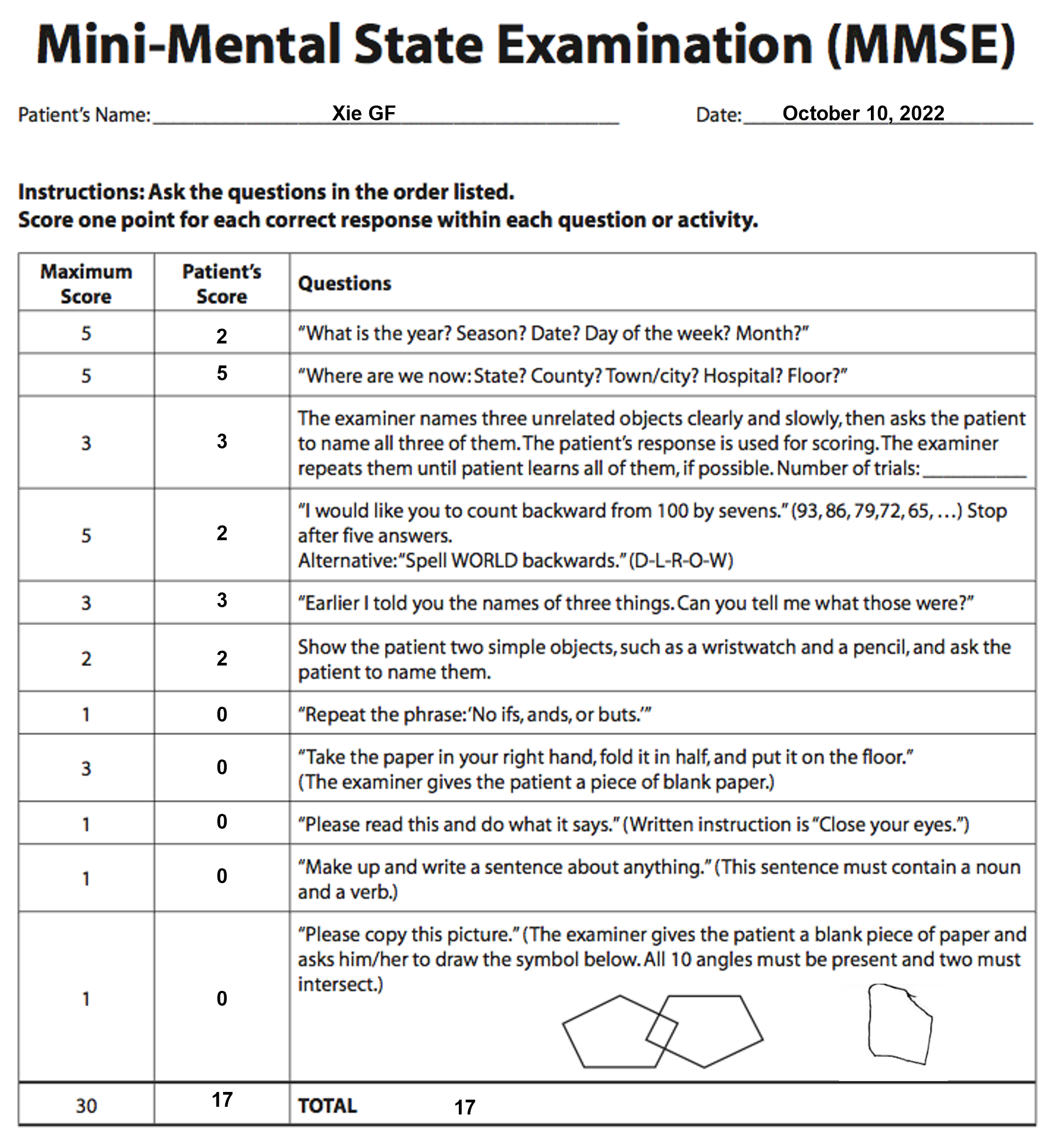

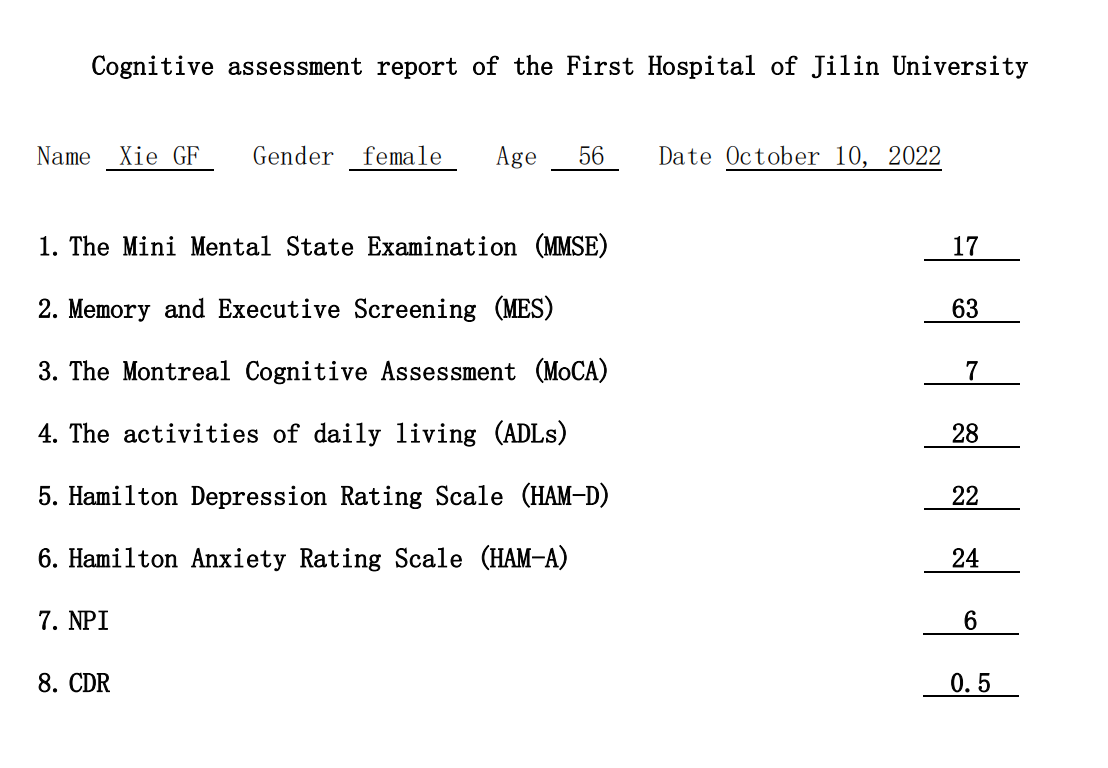


**Figure S2.** After 1 month, the patient's cognitive impairment improved significantly before teaching, and the MMSE recovered to 17 points(MMSE:17;MES:63;MOCA:7;ADL:28;HAMD:22;HAMA:24;NPI:6;CDR:0.5)


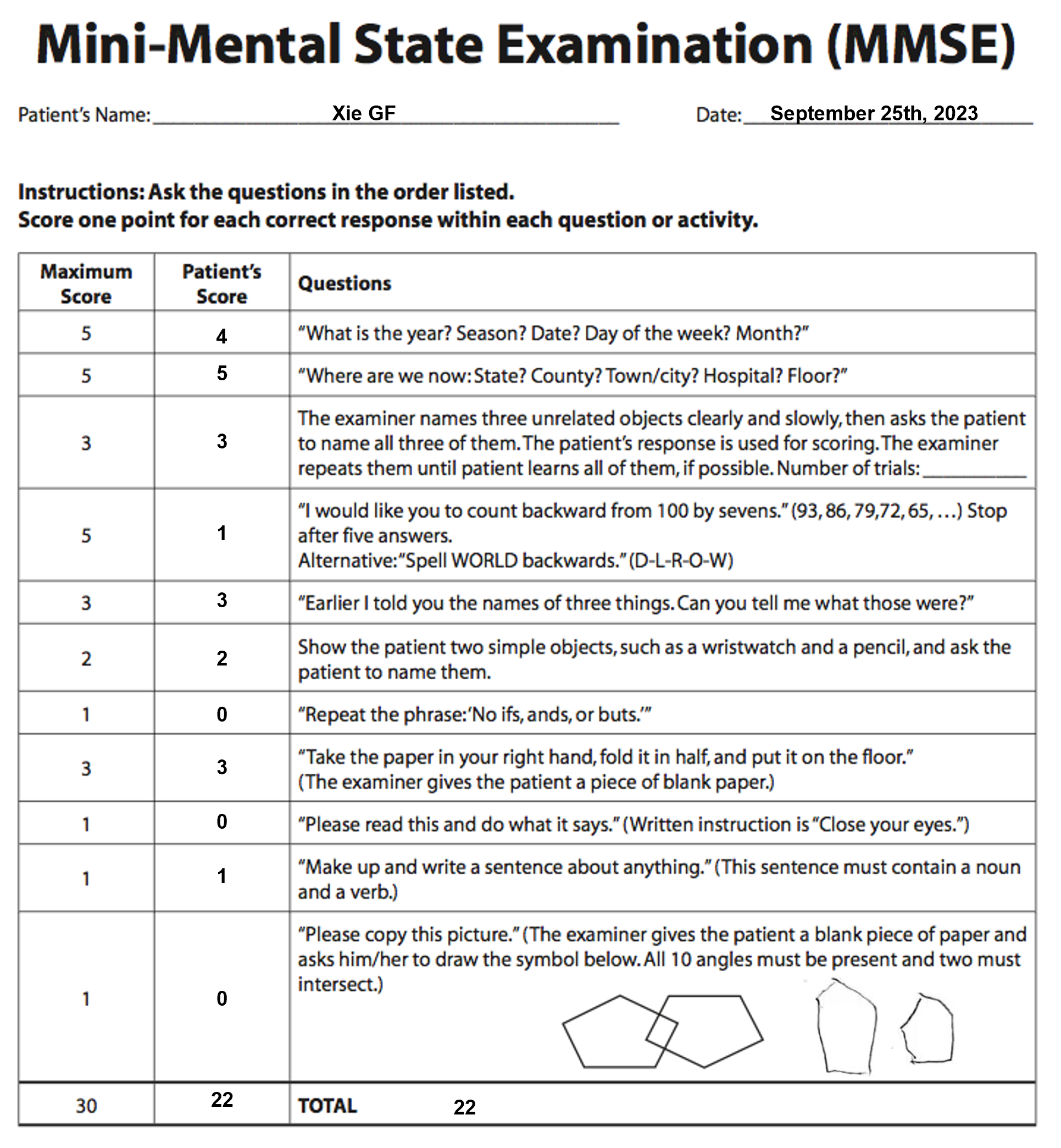

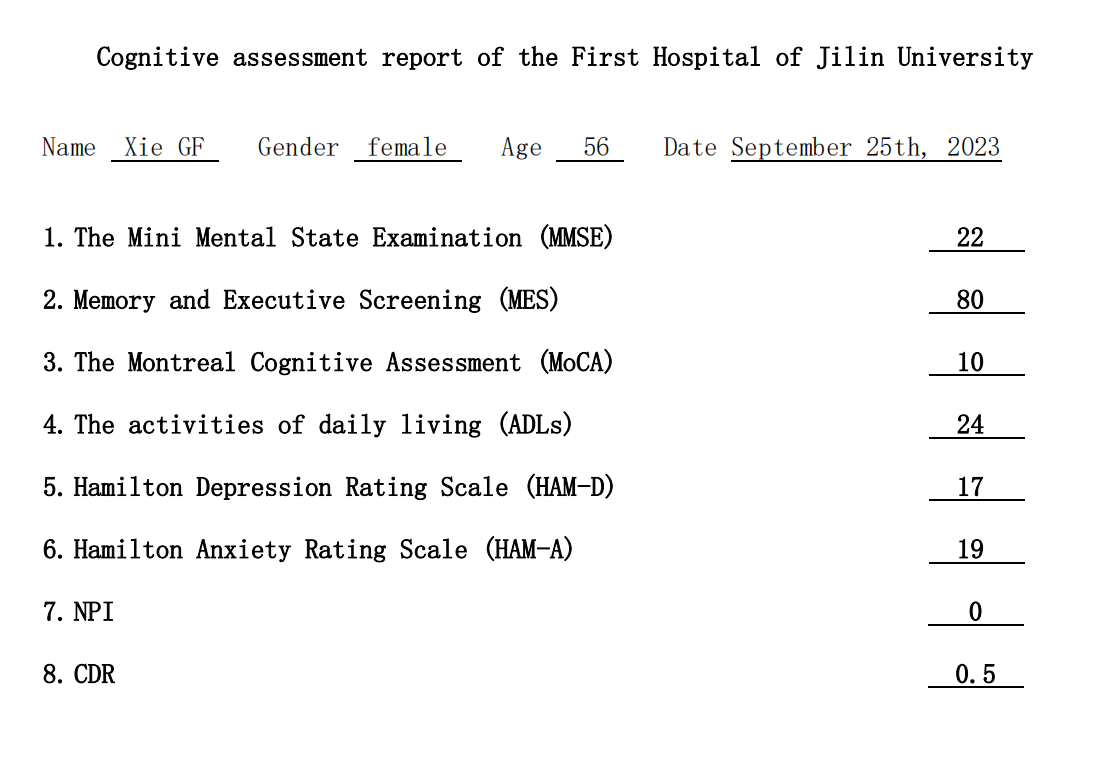


**Figure S3.**One year later, the patient's cognitive level had completely recovered, the MMSE had recovered to 22 points(MMSE:22;MES:80;MOCA:10;ADL:24;HAMD:17;HAMA:19;NPI:0;CDR:0.5)
